# Efficient Breeding by Genomic Mating

**DOI:** 10.3389/fgene.2016.00210

**Published:** 2016-11-29

**Authors:** Deniz Akdemir, Julio I. Sánchez

**Affiliations:** ^1^Plant Breeding and Genetics, Cornell UniversityIthaca, NY, USA; ^2^School Of Agriculture and Food Science Agriculture and Food Science, University College DublinDublin, Ireland

**Keywords:** breeding, complex traits, genomic selection, phenotypic selection, genome-wide markers

## Abstract

Selection in breeding programs can be done by using phenotypes (phenotypic selection), pedigree relationship (breeding value selection) or molecular markers (marker assisted selection or genomic selection). All these methods are based on truncation selection, focusing on the best performance of parents before mating. In this article we proposed an approach to breeding, named genomic mating, which focuses on mating instead of truncation selection. Genomic mating uses information in a similar fashion to genomic selection but includes information on complementation of parents to be mated. Following the efficiency frontier surface, genomic mating uses concepts of estimated breeding values, risk (usefulness) and coefficient of ancestry to optimize mating between parents. We used a genetic algorithm to find solutions to this optimization problem and the results from our simulations comparing genomic selection, phenotypic selection and the mating approach indicate that current approach for breeding complex traits is more favorable than phenotypic and genomic selection. Genomic mating is similar to genomic selection in terms of estimating marker effects, but in genomic mating the genetic information and the estimated marker effects are used to decide which genotypes should be crossed to obtain the next breeding population.

## 1. Introduction

Selection is an evolutionary phenomenon that affects the phenotypic distribution of a population. From a breeding point of view, truncation selection means breeding from the best individuals (Falconer et al., 1996). Breeders have been selecting on the basis of phenotypic values since domestication of plants and animals; this is called phenotypic selection (PS). More recently, breeders have substantially used the pedigree-based prediction of breeding values (BV's) for the genetic improvement of complex traits (Henderson, 1984; Gianola and Fernando, 1986; Crossa et al., 2006; Piepho et al., 2008); which is refereed to as breeding value selection.

Since the invention of the polymerase chain reaction by Mullis in 1983, the enhancements in high throughput genotyping (Lander et al., 2001; Margulies et al., 2005; Metzker, 2010) have transformed breeding pipelines through marker-assisted selection (MAS) (Lande and Thompson, 1990), marker assisted introgression (Charcosset and Hospital, 1997), marker assisted recurrent selection (Bernardo and Charcosset, 2006), and genomic selection (GS) (Meuwissen et al., 2001; Isidro et al., 2016). The latter use genome-wide markers to estimate the effects of all genes or chromosome positions simultaneously (Meuwissen et al., 2001) to calculate genomic estimated BV's (GEBVs), which are used for selection of individuals. This process involves the use of phenotypic and genotypic data to build prediction models that would be used to estimate GEBV's from genome wide marker data. It has been proposed that GS increases the genetic gains by reducing the generation intervals and also by increasing the accuracy of estimated BV's. However, many factors are involved in the relative per unit of time efficiency of GS and its short and long time performance (Daetwyler et al., 2007; Jannink et al., 2010).

Some optimized parental contribution calculation schemes have been proposed to balance the gain from selection and average inbreeding and co-ancestry (Wray and Goddard, 1994; Brisbane and Gibson, 1995; Meuwissen, 1997; Meuwissen et al., 2001; Sonesson et al., 2012; Clark et al., 2013). Approaches that seek for an optimal subset of mates among potential male and female candidates have been formulated from an animal breeding perspective in (Allaire, 1980; Jansen and Wilton, 1985; Kinghorn, 1998) and in subsequent articles (Kinghorn and Shepherd, 1999; Fernández et al., 2001; Berg et al., 2006; Kinghorn, 2011; Pryce et al., 2012; Sun et al., 2013). Researchers (Kinghorn and Shepherd, 1994; Hayes et al., 1998; Shepherd and Kinghorn, 1998) have used mate selection to maximize predicted merit of progeny in a simulated animal breeding scenarios. The mate selection in these articles involved two components (i) a mate selection index (MSI), (ii) a mate selection algorithm to be used to find the mating set which maximizes the MSI. These strategies were termed look ahead mate selection (LAMS) schemes, as they involved mate selection among predicted progeny (Hayes et al., 1998), and they encompass consideration of within-cross variance (Shepherd and Kinghorn, 1998). Although there are great parallels between these works and the current one, the MSI in these papers used a pedigree based co-ancestry matrix and additive genetic value estimates based on this matrix and observed phenotypes. The novelty in this article was the use of genomic data in terms of markers to obtain estimates of BV's, estimates of Mendelian effects, and co-ancestries and incorporate them into a index; the marker effects that are used in these calculations were estimated with a marker based regression model of the estimated genetic values of the individuals as in GS.

**Figure 1:**
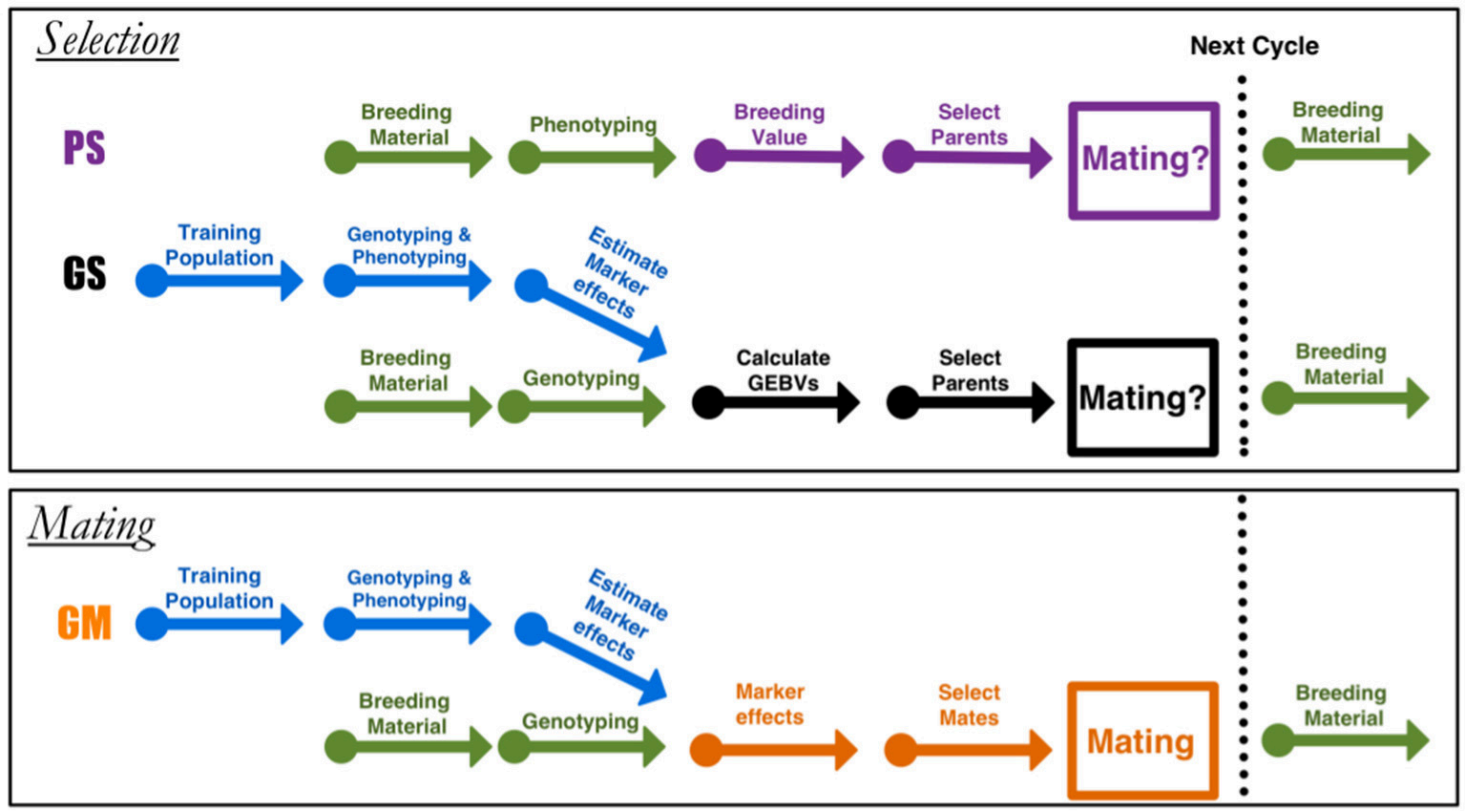
**Diagram for the different breeding approaches**. Phenotypic selection (PS) and genomic selection (GS) are truncation selection methods, and genomic mating (GM) is the mating approach. Arrows indicate the different stages in a breeding cycle. In PS, starting with a set of parents as breeding material, selection is performed based on phenotypes.

**Figure 2:**
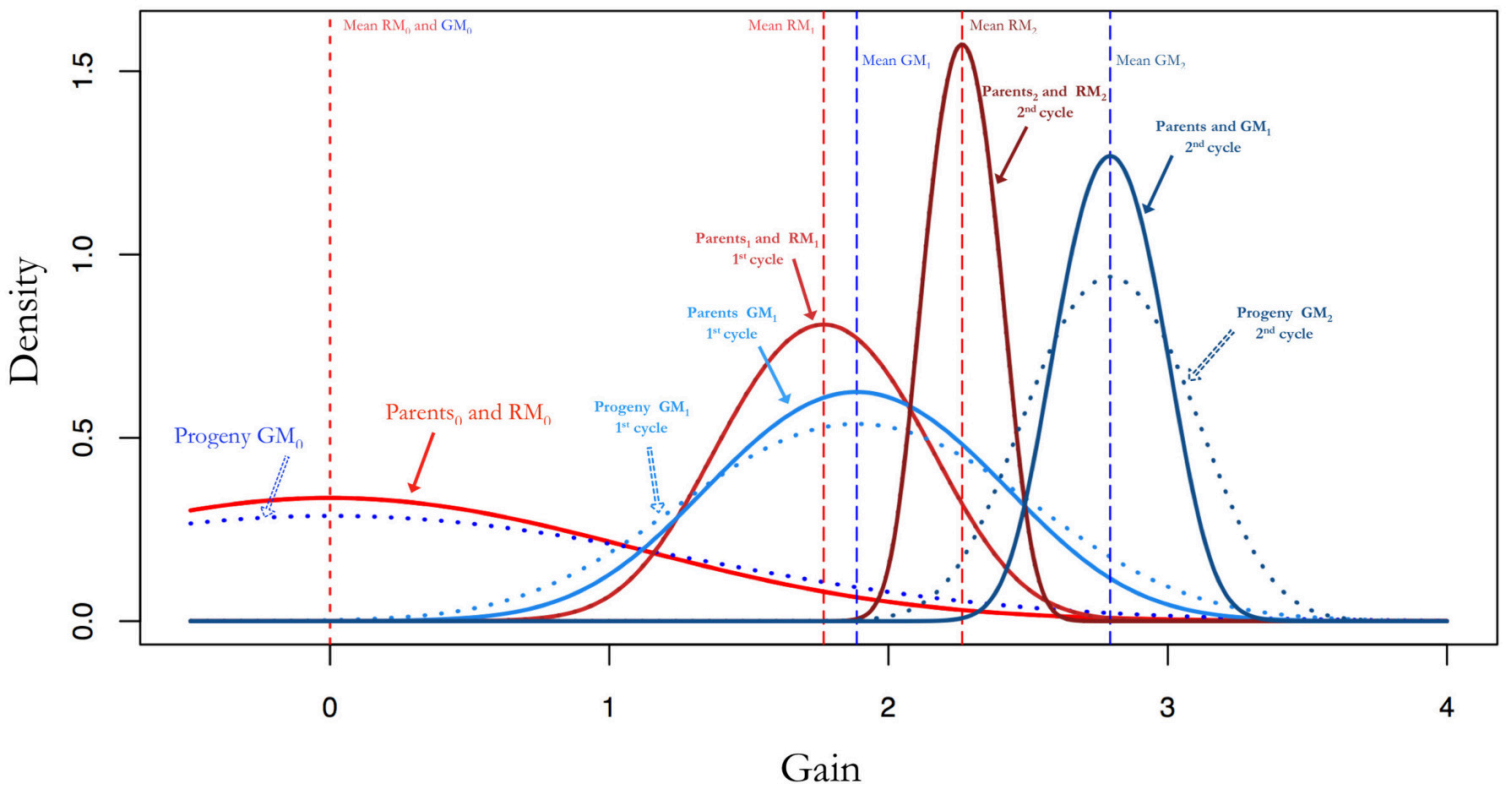
**Additive genetic variance and gains by selection after random mating compared to gains by selection after random mating optimal mating**. The parents and progeny for the random mating populations have the same allele frequencies and therefore the same additive genetic variance. If parents are mated with optimal mating the result is an increase in the additive genetic variance. This is pronounced as larger gains and maintains additive genetic variability for as the breeding progresses.

Breeders have used marker assisted breeding to stack genes using complementary crosses when the trait of interest is regulated by only a few loci. For complex traits, on the other hand, there is a scarcity of methods available to breeders. Both of PS and GS focus on improvement by truncation selection, mainly ignoring the role of mating and complementation as an evolutionary force (Figures 1, 2). For this reason both PS and GS are, in a sense, inefficient for improving complex traits in the long term. Methods that seek only a balance between genetic gains and inbreeding are incomplete because they ignore the variances in the genetic values; measures of gain do not completely capture the full potential of a mate pair.

This paper proposes genomic mating (GM) as an alternative to GS. Genomic selection focuses on best performance of parents before mating, while GM includes information on complementation of parents to be mated and thereby is more sustainable in the longer term (Figure 1). Our method is inspired by the GS and the classical mate selection based on pedigrees (Kinghorn, 1998; Kinghorn and Shepherd, 1999; Kinghorn, 2011). In the remaining of this article, we assume that a high density marker data is available for the current breeding population from which the co-ancestry coefficients can be calculated, and that there is no pedigree information. In view of the reducing genotyping costs, mostly incomplete or non-existing pedigrees in plant populations, this assumption is a reasonable one.

## 2. Methods

It is widely accepted that short term gains from selection increases with increased selection intensity. However, increasing selection reduces the genetic variability, which increases the rates of inbreeding and co-ancestry and may reduce gains in the long term run. Most of the selection in plant breeding are designed to maximize genetic gain but some approaches try to balance the gain from selection and variability. We will give a brief review of these approaches since they relate to the mating theory.

### 2.1. Current methodology

Many authors (Goddard, 2009; Jannink, 2010; Sonesson et al., 2012; Clark et al., 2013; Sun et al., 2013) have expressed the importance of reducing inbreeding in PS and GS for long-term success. They argued that GS is likely to lead to a more rapid decline in the selection response unless new alleles are continuously added to the calculation of GEBVs, stressing the importance of balancing short and long term gains by controlling inbreeding in selection.

Let *A* be a matrix of pedigree based numerator relationships or the additive genetic relationships between the individuals in the genetic pool (this matrix can be obtained from a pedigree of genome-wide markers for the individuals) and let *c* be the vector of proportional contributions of individuals to the next generation under a random mating scheme. The average inbreeding and co-ancestry for a given choice of *c* can be defined as $r=\frac{1}{2}c^{\prime }Ac.$ If *b* is the vector of GEBV's, i.e., the vector of BLUP estimated BV's of the candidates for selection. The expected gain is defined as *g* = *c*′*b*. Without loss of generality, we will assume that the breeder's long term goal is to increase the value of *g*.

In Wray and Goddard (1994), Brisbane and Gibson (1995), and Meuwissen (1997) an approach that seeks minimizing the average inbreeding and co-ancestry while restricting the genetic gain is proposed. The optimization problem can be stated as

(1) $$\begin{array}{l} \underset{c}{\text{minimize}}\;r=c^{\prime }\frac{A}{2}c\\ \text{subject to }c^{\prime }b=\rho \\ \;c^{\prime }1=1\\ \;c\geq 0, \end{array}$$

where ρ is the desired level of gain.

This problem is easily recognized as a Quadratic optimization problem (QP). There are many efficient algorithms that solves QP's so there is in practice little difficulty in calculating the optimal solution for any particular data set. Recently, several allocation strategies were tested using QP's in (Goddard, 2009; Schierenbeck et al., 2011; Pryce et al., 2012). It is easy to extend these formulations to introduce additional constraints as positiveness, minimum-maximum for proportions, minimum-maximum for number of lines (cardinality constraints).

Some authors recommended mate selection approaches that also seek a balance between gain and inbreeding from an animal breeding perspective (Allaire, 1980; Jansen and Wilton, 1985; Kinghorn, 1998). Simple methods such as sequential selection (Pryce et al., 2012) or linear programming (LP) (Jansen and Wilton, 1985; Weigel and Lin, 2000) have been used to find mate designs to avoid more-related pairs and find less-related pairs. Some approaches example the use of genome-wide marker information (Fernández et al., 2001; Pryce et al., 2012; Sun et al., 2013), but these approaches mainly deal with the restricted case of mating after evaluation of breeding population and selection. Kinghorn, in a series of articles (Kinghorn, 1998; Kinghorn and Shepherd, 1999; Kinghorn, 2011), describes an algorithmic approach that separates the optimization and the objective function for the mate selection approach and therefore can be used for a wide array of optimization criteria (MSI) with hard and soft constraints. The MSI recommended by Kinghorn (1998) involves calculation of the merit of potential calves as half of the sire's breeding value plus half the dam's breeding value minus a weighted penalty on the estimated progeny inbreeding coefficient. The method in Pryce et al. (2012) uses a similar MSI based on phased haplotypes. These sources also introduce a number of constraints related to constraints on reproduction or resources. The optimization criteria in these papers have the same flavor as the quadratic optimization problem in Equation (1) and is expected to give similar solutions with the parental contributions approach for large breeding populations. We agree with these previously recommended approaches in the sense that mate selection is an optimization problem. However, we use markers, genomic prediction models and estimates of these models from phenotypic experiments (as the training data becomes available) and include a genomically estimated additive variance term for each mate pair; therefore the optimization problem in the recent paper is a related but different one; for instance, our criterion is neither linear nor quadratic.

By solving the QP in Equation (1) for varying values of ρ, or using the similar criteria in the mate selection approaches, we can trace out an efficient frontier curve, a smooth non-decreasing curve that gives the best possible trade-off of genetic variance against gain. This curve represents the set of optimal allocations and it is called the efficiency frontier (EF) curve in finance (Markowitz, 1952) and breeding literature.

The implementation of PS in our simulations did not use any genotypic information or pedigrees. Basically, it referred to selecting the individuals with best observed phenotypes to be parents in the next generation. Results elsewhere (Forni et al., 2011) indicate that there would be no significant differences between PS and GS if a pedigree from many generations is used in pedigree based estimation of BV's. In addition, there are methods to combine pedigrees with marker based relationship matrices (Legarra et al., 2009; Meuwissen et al., 2011) which would result in a yet another selection approach.

Based on the selection intensity the best individuals were determined in each step based on their phenotypic values and a random mating was employed among these individuals to determine the mates. On the other hand, GS used GEBVs obtained from models that are updated by phenotypic values every two cycle using only the most current genotypic and phenotypic data. The selection of parents in each cycle was done according to the GEBVs and the selection intensity. The mate assignment was at random as in PS. Efficient GS referred to the selection of parents in each cycle using the optimal parental contribution proportions obtained by solving the optimization problem in Equation (1). The specific solution in each cycle was selected among the Pareto optimal solutions along the EF curve by setting ρ to the 90th percentile of the GEBVs in the current population (we have decreased ρ if a gradually if the solution was dominated by a single parent). The mates for next cycle were assigned using the optimal contribution proportions.

### 2.2. Optimal genomic mating

There are several alternative measures of inbreeding based on mating plans (Leutenegger et al., 2003; Wang, 2011). In this article, we have used a measure derived under the infinitesimal genetic effects assumption proposed by Quaas (1988) and Legarra et al. (2009). A measure of gain, i.e., the total expected breeding value of the progeny, can also be calculated from the results of the same authors (Quaas, 1988; Legarra et al., 2009). However, in our belief, the expected value by itself is not a good measure of possible gains since it carries no information about the variability of BV's among full-sibs. Therefore, we have derived a measure called the risk of a mating plan (this is related to the concept of “usefulness”) by increasing the expected of the progenies by a small amount (the intensity is controlled by the parameter λ_1_) proportional to their expected variance (standard deviation) calculated under the infinitesimal effects assumption.

Combining the measures of inbreeding and risk into one leads to the formulation of the mating problem:

(2) $$\begin{array}{l} \underset{P_{32}}{\text{minimize}}\;r\left(\lambda_{1},\lambda_{2},P_{32}\right)=-Risk\left(\lambda_{1},P_{32}\right)\;+\;\lambda_{2}\;*\;Inbreeding\left(P_{32}\right) \end{array}$$

where λ_2_ ≥ 0 is the parameter whose magnitude controls the amount of co-ancestry in the progeny, and the minimization is over the space of the mating matrices *P*_32_ construction of which is described in detail below. λ_1_ controls allele heterozygosity weighted by the marker effects and λ_2_ controls allele diversity. When λ_1_ = 0 the risk measure is the same as total expected gain.

Now, we give the details of how the measures *Risk*(λ_1_) and *Inbreeding* are defined in this paper. First, we make the following assumptions: (a) Diploid behavior at meiosis, (b) Uncorrelated genes distribution, (c) Absence of allelic interactions, (d) No multiple alleles at those loci controlling the character, (e) No of genotype-environment interaction.

Let $b={\left(b_{1}^{\prime },b_{2}^{\prime },b_{3}^{\prime }\right)}^{\prime }$ denote the vector of genetic effects corresponding to the parents and progeny, where *b*_1_ and *b*_2_ are the genetic effects of the *N* parents and *b*_3_ are the genetic effects of the *N*_*c*_ progeny. Let the pedigree based numerator relationship matrix for the individuals in *b* be *A* and this matrix is partitioned as

$$A=\left[\begin{array}{ccc} A_{11} & A_{12} & A_{13}\\ A_{21} & A_{22} & A_{23}\\ A_{31} & A_{32} & A_{33} \end{array}\right]$$

corresponding to the partitions of *b*. Suppose, we also have the markers for the parents in the second partition, and *u*_2_ = *Ma* where *M* is the matrix of minor allele dosages, coded as 0, 1, and 2. Let *M*_*c*_ be the *N* × *m* marker allele frequency centered incidence matrix and *a* be the vector of marker effects. Variance-covariance of *b*_2_ can be written as

$$Var\left(b_{2}\right)=\sigma_{b}^{2}\frac{M_{c}M_{c}^{\prime }}{k}=\sigma_{b}^{2}G$$

with *G*, the genomic relationship matrix, defined as $\frac{M_{c}M_{c}^{\prime }}{k}$ where $k=\overset{m}{\underset{j=1}{\sum }}2p_{j}\left(1-p_{j}\right)$ is twice the sum of heterozygosities of the markers (VanRaden, 2008).

Following Quaas (1988) and Legarra et al. (2009), let *P* be a matrix containing the transitions from ancestors to offspring. We will refer *P* as the mating or parentage matrix. Then, we can write *b* = *Pb* + **ψ** where **ψ** is the vector of Mendellian samplings and founder effects with a diagonal variance D. In particular, using only the rows of *P* corresponding to the *b*_3_ the relationship is written as

$$b_{3}=[\begin{array}{ccc} P_{31} & P_{32} & P_{33} \end{array}]\;\left[\begin{array}{c} b_{1}\\ b_{2}\\ b_{3} \end{array}\right]+\Psi_{3}$$

which can also be stated as a regression equation of the form $b_{3}={\left(I-P_{33}\right)}^{-1}\left(P_{31}b_{1}+P_{32}b_{2}+\psi_{3}\right)$ (Quaas, 1988). The variance-covariance matrix of *b*_3_ is given by

(3) $$\begin{array}{l} Var(b_{3})={\left(I-P_{33}\right)}^{-1}(P_{31}A_{11}P_{31}^{\prime }+P_{32}{GP}_{32}^{\prime }+P_{32}A_{21}P_{31}^{\prime }\\ \;+\;P_{31}A_{12}P_{32}^{\prime }+D_{3}){\left(I-P_{33}\right)}^{\prime -1}. \end{array}$$

The variances caused by Mendelian sampling in *D*_3_ are related to inbreeding in the parents via

$$var\left(\psi \right)\propto \left(1/2-\left(F_{1}+F_{2}\right)/4\right)$$

where *F*_1_ and *F*_2_ are the inbreeding coefficients of the two parents which can be extracted from the diagonals of G. The variance-covariance formula reduces to

$$Var\left(b_{3}\right)=P_{32}GP_{32}^{\prime }+D_{3}$$

if all the founders are genotyped (no *P*_31_), and a relatively simple mating strategy is assumed where founders are the only parents and no back-crossing is allowed (*P*_33_ = 0). This is the assumption made for the remainder of this paper and in this case *P*_32_ is a *N*_*c*_ × *N* matrix (*N*_*c*_ children from *N* parents) with each row having two 1/2 values at positions corresponding to two distinct parents or only a value of 1 at the position corresponding to the selfed parent. All the other elements of this matrix are zero. Nevertheless, one can easily imagine situations where some of the founders are not genotyped or when some of the progeny also have progeny, then the formula in Equation (3) will be relevant. If some founders are not genotyped but a pedigree is available relating them to the rest of the founders then the variance-covariance for the founders, *Var*(*b*_1_, *b*_2_), can be calculated using the relationship matrix in Legarra et al. (2009). Furthermore, construction of the mating matrices for more complex mating plans is described in Quaas (1988).

*Var*(*b*_3_) gives us the expected variance-covariance of the progeny given the mating matrix *P*_32_ and the realized relationship matrix *G* of the parents. This can be used as to measure the expected genetic diversity of a mating plan: we can use a measure in line with the inbreeding term in Equation (1) by

(4) $$\begin{array}{l} Inbreeding\left(P_{32}\right)=1_{N_{c}}^{\prime }Var\left(b_{3}\right)1_{N_{c}}=1_{N_{c}}^{\prime }\left(P_{32}GP_{32}^{\prime }+D_{3}\right)1_{N_{c}}. \end{array}$$

We also need a measure for genetic gain. A simple measure of gain for a given mating plan expressed in *P*_32_ can be constructed from the expected value of *b*_3_:

$$E\left(b_{3}\right)=P_{32}Ma$$

and an overall measure can be written as

$$Gain\left(P_{32}\right)=1_{N_{c}}^{\prime }E\left(b_{3}\right).$$

Finally, we want to complement the measure “gain” with a measure of within cross-variance for the genetic levels of children of the parent pairs. Suppose the organism under study is diploid. We can re-code the markers matrix *M* coded as −1, 0, and 1 into a *N* × *m* matrix M^*^ using the information in the marker effects vector *a* such that markers are coded as the number of beneficial alleles as 0, 1, or 2. This is achieved by first obtaining marker effects estimates and then using the sign of the estimates to determine what is a beneficial allele. We can also obtain a related marker effects vector a^*^ by replacing the original marker effects by the effects of the beneficial alleles (a^*^ = |*a*|) so that we have $Ma=\left(M^{*}-1_{N\times m}\right)a^{*}.$ For a given parent pair, we can calculate the vector expected number of beneficial alleles of the children of these parents using a transition vector *p* as **μ** = *E*(*m*) = *p*′M^*^. In addition, for each locus we can calculate the variance for the number of beneficial alleles from the number of alleles the parents have and put them in a vector which we will denote by **σ**_*p*_ = (σ_*p*1_, σ_*p*2_, …, σ_*pm*_). Calculation of elements of **σ**_*p*_ from the coding in M^*^ can be as in Table 1. We define risk measure for this parent pair as

$$Risk(\lambda_{1})=(p^{\prime }M^{*}+\lambda_{1}*\left(\begin{array}{c} \sqrt{\sigma_{p1}}\\ \sqrt{\sigma_{p2}}\\ \ldots \\ \sqrt{\sigma_{pm}} \end{array}\right)-1_{m}{)}^{\prime }a^{*}$$

where λ_1_ ≥ 0 is the risk parameter and *m* is the number of markers. The risk of a mating plan (which is expressed in *P*_32_) is the sum of all the risk scores for all mate pairs in that plan which we will denote by *Risk*(*P*_32_, λ_1_).

**Table 1:** **Expected values and variances**.

**Parent 1**	**Parent 2**	**Expected number of beneficial alleles**	**Variance of number of beneficial alleles**
1	1	2	0
1	0	1.5	0.25
0	1	1.5	0.25
1	−1	1	0
−1	1	1	0
0	0	1	0.5
0	−1	0.5	0.25
−1	0	0.5	0.25
−1	−1	0	0

*Calculation of mean number and variance of the beneficial alleles of progeny at each locus from the beneficial allele code (−1, 0, 1) of the parents at the same locus*.

If the risk parameter λ_1_ is set to zero then we have

$$Risk\left(P_{32},\lambda_{1}=0\right)=1_{N_{c}}^{\prime }E\left(b_{3}\right)=1_{N_{c}}^{\prime }P_{32}Ma.$$

The magnitude of λ_1_ is related to the desire of the breeder to take advantage of within cross variances and encourages mating parents that are heterozygotes at quantitative trait loci (QTL).

In this sense, the efficient mating problem can be stated as an optimization problem as follows:

(5) $$\begin{array}{l} \begin{array}{cc} \underset{P_{32}}{\text{minimize}} & Inbreeding\left(P_{32}\right)=1_{N_{c}}^{\prime }\left(P_{32}GP_{32}^{\prime }+D_{3}\right)1_{N_{c}}\\ \text{subject to} & \begin{array}{c} Risk\left(P_{32},\lambda_{1}\right)=\rho . \end{array} \end{array} \end{array}$$

In the above optimization problem, we are trying to minimize the inbreeding in the progeny while the risk is set at the level ρ ≥ 0. In the remainder of this paper, we will use the the equivalent formulation of the mating problem in Equation (2).

The risk measure for each mate pair is calculated as the sum of the estimated breeding value for the progeny plus λ_1_ times the expected standard deviation of the BV's of the progenies of this pair. When λ_1_ is set to zero, only the expected BV's for the progenies are taken into account. If λ_1_ > 0, then the variation expected in the progenies enters the picture and the pairs that have higher expected variance among their progenies will have higher risk values among mate pairs that have same expected breeding value. Keeping everything else constant the effect of an increased variance for the progenies of a mate pair is increasing the chances that one of the progenies of this mate pair will have a high breeding value compared to the mean value of the progenies. For instance, if we assume a normal distribution for the breeding value of the progenies of a mate pair, about 16% of the progeny will have breeding value higher than one standard deviation above the mean, i.e., mate pairs that have high variance have the potential to produce progenies with high BV's. However, since the distribution is symmetric around the mean, the same mate pair will have equal chances of producing progenies with low BV's. Therefore, we named our measure as “risk”. We note also that other measures of expected variance have been proposed (Zhong and Jannink, 2007) and these can be used instead of the one we have proposed above. For example, it is possible to calculate this variance by simulating progenies and their expected BV's for parent pairs, and one can easily include information about the LD in these simulations. Our choice of the measures of inbreeding and risk are driven by computational efficiency in order to keep the optimization over the mates feasible. In the next section, we give some examples of GM; we provide a procedural form of the GM algorithm used in these examples by Procedure 1.

**Procedure 1:** Genomic Mating

1: **for** Each breeding cycle *i* ∈ (0, 1, 2, 3, …) **do**
2: Let *P*_*i*_ denote the *i*th population of parents, *M*_*i*_, *G*_*i*_ be corresponding markers matrix, the matrix of coancestories.
3: **if** *i* is even **then**
4: obtain the vector of estimated genetic values ${\hat{g}}_{i}$ for *P*_*i*_ based on a phenotypic experiment,
5: based on a regression of the estimated genetic values $\hat{g}$ on the additive coding of the markers estimate the vector of additive effects for the markers; ${\hat{b}}_{i}.$
6: **else**
7: ${\hat{b}}_{i}={\hat{b}}_{i-1}.$
8: Given *N_c_*, λ_1_ and λ_2_ find the optimizer of Equation (5) using the GA described in Procedure 2, denote this by $P_{32}^{i}.$
9: Make the crosses indicated by to $P_{32}^{i}$ using parents *P*_*i*_ to obtain *P*_*i*+1_.

The differences between the expressions in Equation (1) (which can be written in the current context by replacing *A* with *G* as *c*′*Gc*) and the current criterion in Equation (2) should be quite noticeable: *c* is the vector of parental contribution proportions and *P*_32_ is the mating matrix that leads to progeny and this reflects the shift of focus to selection of mates rather than providing only proportions but leaving the mating problem unanswered. Given *P*_32_ proportion of parental contributions can be calculated, however there is no way of obtaining an optimal *P*_23_ from the knowledge of *c*. This focus shift also leads to the introduction of the diagonal matrix *D*_3_ for the variances in progeny caused by Mendelian sampling of the alleles in the parents in the latter. In addition, a representation of the criterion in Kinghorn (Kinghorn, 1998) using the current notations is given by $MSI=1_{N_{c}}^{\prime }P_{23}GP_{23}^{\prime }1_{N_{c}}+\lambda 1_{N_{c}}^{\prime }P_{23}b.$ Writing ${\hat{c}}^{\prime }=1_{N_{c}}^{\prime }P_{23}$ and $MSI={\hat{c}}^{\prime }G\hat{c}+\lambda {\hat{c}}^{\prime }b,$ we can see that this and Equation (1) have the same form and since we can expect these criterion to give similar results if *N*_*c*_ is large and no other constraints were imposed.

As compared to the quadratic optimization problem in Equation 1 which admits easy solutions, the optimization problem in Equation (2) is a combinatorial problem whose order increases with the number of individuals in the breeding population and the number of progeny. The list of all possible mates including self-mates is a list of length *N*_*p*_(*N*_*p*_ + 1)/2. The problem can be stated as selecting *N*_*c*_ elements with replication from this list. The number of un-ordered *N*_*c*_-tuples of an *N*_*p*_(*N*_*p*_ + 1)/2-set is ${\left(N_{p}\left(N_{p}+1\right)/2\right)}^{N_{c}}/N_{c}!.$ We have devised a genetic algorithm to find good solutions for this optimization problem in reasonable computing time. The optimization procedure that is used in optimal mating problem is a modified genetic algorithm (GA) supplemented with tabu search (See Procedure 2).

**Procedure 2:** Genetic Algorithm

1: lnitialization - Create an initial population of solutions of desired size, *S*_0_. The population of solutions denoted by the capital letter *S* are sets, elements of which are particular mating designs (a list of *N*_*c*_ mate pairs).
2: *t* = 0.
3: Memory for tabu is empty, *MemTabu*_*t*_ = *NULL*;
4: **repeat**
5: *t* = *t* + 1,
6: Evaluation -For each solution in *S*_*t*−1_ calculate the criterion value in Equation (5),
7: Selection - Identify the best solutions by the ordering of criterion values, these are denoted by *E*_*t*_,
8: Elitism- Let the best solution in *E*_*t*_ be *s*_*t*_. Put *s*_*t*_ in *S*_*t*_,
9: Tabu- Update memory for tabu by letting *MemTabu*_*t*_ = *S*_*t*−1_.
10: **repeat**
11: Crossover-Randomly pick two solutions in *E*_*t*_. Recombination of these two solutions are obtained by summing the frequency distributions of these solutions and sampling with new solutions using probabilities corresponding to this combined frequency distribution.
12: Mutation - With a given probability decrease the frequency of a mate that has positive frequency by some integer value less than the current frequency of that mate and increase the frequency of some other mate pair is by the same amount.
13: **if** the resulting solution is in *MemTabu*_*t*_ **then**
14: eliminate solution
15: **else**
16: Insert solution into *S*_*t*_.
17: **until** *S_t_* has *N_pop_* solutions.
18: **until** Convergence is reached
19: Evaluation -For each solution in *S*_*t*_ calculate the criterion value in Equation (5),
20: Selection - Identify the best solutions by the ordering of criterion values, these are denoted by *E*_*t*_,
21: Elitism- Let the best solution in *E*_*t*_ be *s*_*t*_. Return *s*_*t*_.

Genetic algorithms (Holland, 1973; Davis, 1991; Goldberg, 2006) are particularly suitable for optimization of combinatorial problems. The idea is to use a population of candidate solutions that is evolved toward better solutions. At each iteration of the algorithm, a fitness function is used to evaluate and select the elite individuals and subsequently the next population is formed from the elites by genetically motivated operations like crossover and mutation. Tabu search is a search where most recently visited solutions are avoided by keeping a track of the previously tried solutions. This avoids many function evaluations and decreases the number of iterations till convergence, it is especially useful for generating new solutions toward convergence.

Each point in the solution space of the optimal mating problem can described by a frequency distribution over the the set of all possible mate pairs. In our case, the recombination of two solutions in this GA are obtained by summing the frequency distributions of these two solutions and sampling with probabilities corresponding to this combined frequency distribution. With a given probability of mutation, a mutation event follows the crossover event and is achieved by decreasing the frequency of a mate that has positive frequency by some integer value less than the current frequency of that mate and increasing the frequency of some other mate pair by the same amount. In our algorithm, the average magnitude of change that is allowed during mutation is called mutation intensity.

It should be noted that the solutions obtained by any GA or any other evolutionary algorithm may be sub-optimal and different solutions can be obtained given a different starting population. Another layer of safety is obtained if the algorithm is started from multiple initial populations and an island model of evolution is used where separate populations are evolved independently for several steps and then the best solutions from these algorithms becomes the initial solutions to evolutionary algorithm, this strategy is also partially employed.

We included the minimum number of parents as a parameter: “minparents” in long term simulations using GM. This allowed us to run the simulations many times without interference. Nevertheless, a better approach in practical situations would be to plot the whole frontier surface and select a solution that has a good risk to diversity ratio.

## 3. Results

**Figure 3:**
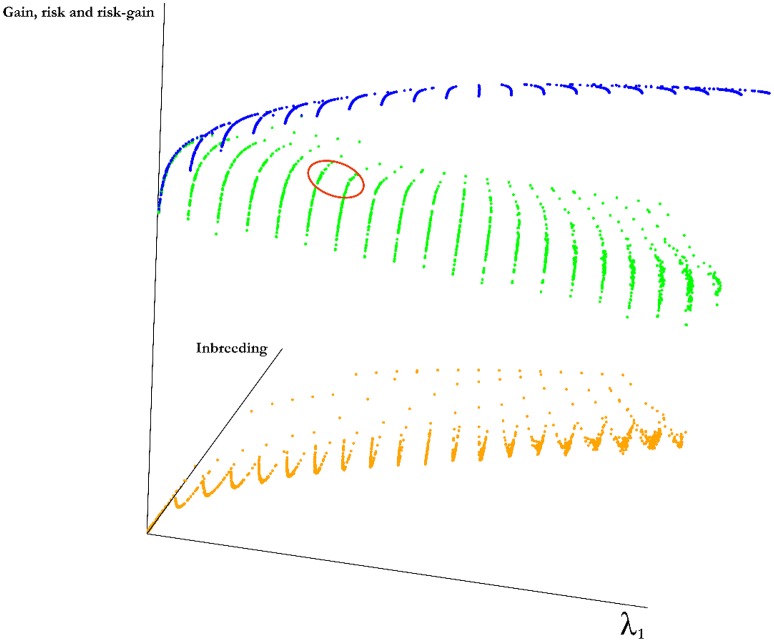
**Frontier surface for a simulated population**. A marker data was created for 50 genotypes by randomly generating 1000 markers for each genotype. By introducing independent and identically normally distributed marker effects at 500 of randomly selected the loci we have defined a trait. Three surfaces are given in the figure. The blue surface represents the optimal values of the objective function in Equation (2). Points below this surface correspond to sub-optimal regions and points above this surface are unattainable. The points along the surfaces are the optimal points balancing gain, risk and inbreeding. The green surface is the expected average genetic value of the progeny and the orange surface is the value of the cross-variance term, these two surfaces add up to the blue surface. Although, it is not possible to determine a best value for the parameters λ_1_ and λ_2_, a reasonable region for this particular experiment is marked by a red ellipse, this is the region in which the rate of increase in inbreeding per unit gain increases sharply and obtaining additional cross variance after this point requires a large decline in gain.

For a set of 50 simulated lines, we have identified optimal mates for the progeny at changing values of λ_1_ and λ_2_. The frontier surface is drawn using the optimal mating algorithm (Figure 3). The coordinates of the points on the curve are the values of estimated risk, inbreeding and the difference between risk and gain. for the optimal sets of mates. The blue surface represents the optimal values of the objective function in Equation (2). Points below this surface correspond to sub-optimal regions and points above this surface are unattainable. The points along the surfaces are the optimal points balancing gain, risk and inbreeding. The green surface is the expected average genetic value of the progeny and the orange surface is the value of the cross-variance term, these two surfaces add up to the blue surface. By changing λ_1_ and λ_2_ we move on this surface. Since the points on this surface correspond the optimal solutions, the breeder should operate on the surface. The optimal solutions to the mating problem at a few selected values of λ_1_ and λ_2_ is in Figure 3.

Efficient frontier surface is the basis for GM. A feasible mating plan is one that meets specified constraints. The EF surface allows breeders to understand how a mating plan's expected risk vary with the amount of inbreeding. Most breeders will be willing to assume a greater inbreeding for a greater risk. However, breeders differ in the amount of inbreeding they are willing to assume for a given risk. Breeders who are inbreeding averse require lower inbreeding for a given amount of risk than breeders who are risk seekers. It can be seen from Figure 3 that the risk is increasing in a smooth fashion as λ_1_ increases. On the other hand, the corresponding gain decreases at an uneven rate. Therefore, the difference between the risk and gain increases at an uneven rate. A reasonable λ_1_ and λ_2_ combination can be found by locating the solution around the point where the gain slows down increasing as we increase λ_2_ and speeds up decreasing as we increase λ_1_.

An optimal mating scheme can be used to increase within cross variance and to decrease average inbreeding and co-ancestry while attaining a certain genetic gain. This will lead to an increased additive variance in the progeny for a given set of parents when compared to a random mating approach. Since the gains in a breeding population can be mostly attributed to additive variance, this will result in higher expected gains with an optimal mating scheme as can be seen in Figures 2, **5A,B**.

**Figure 4:**
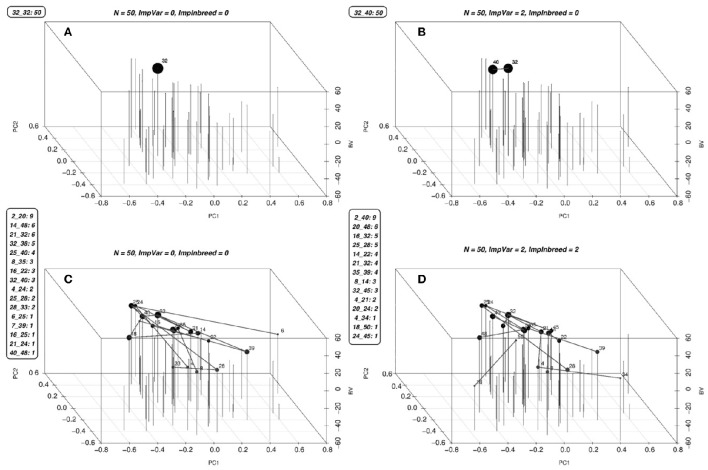
**Optimal solutions for a simulated population**. Optimal solutions to the mating problem at a few selected values of λ_1_ and λ_2_ are in **(A–D)**. The list of mates and the number of crosses for each mate is given along the figures. The first two coordinates are used to display the genetic relationships of the lines using the first two principal components, the third coordinate displays the BV's of the parents. Each parent is represented by a vertical bar. The lines between the vertical bars represent the matings and the size of the points on the bars are proportional to the number of crosses between that parent and any other. Since the mating algorithm is discrete and the number of genotypes contributing to the next generation increase starting from one as we increase the λ_2_, we can identify a point to operate on this surface by slowly increasing the λ_2_ until a desired minimum number of genotypes are included in the solution.

**Figure 5:**
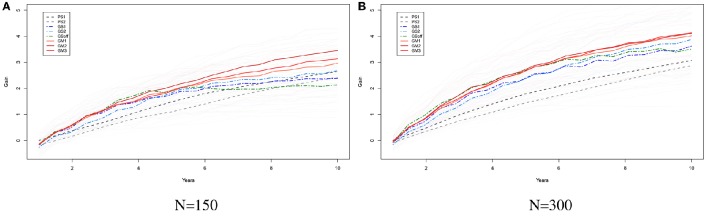
**The long term behavior of PS, GS, Efficient GS and GM**. Starting from 2 founders we have formed a population of 150 **(A)** and 300 **(B)** genotypes with 1000 SNPs at 3 chromosomes each and carried this population through 200 generations of random mating and 100 generations of phenotypic selection based on a complex trait (300 QTL at random locations on each chromosome) with 0.5 heritability generated based on the infinitesimal model. Starting from this initial population, we have simulated 10 rounds of PS, and 20 rounds of GS and GM (assuming one cycle of PS and two cycles of GS and GM per year). For GS and GM, the marker effects were estimated from data once per year. The results of 10 replication of this simulation with selection intensity 10% (PS1, GS1) and 20% (PS2, GS2) for PS and GS; Efficient GS (GSeff); and GM with λ_1_ = 0, 5, 10 (GM1, GM2, GM3). Each thin line represents the genetic gains over cycles by different methods over a replication of the experiment. The thick lines show the mean improvement for each of the methods over 10 replications. In these simulation studies there is a clear advantage of using GM as a breeding method.

Figures 4A,B show the results from simulations for the study of the long term behavior of PS, GS, and GM. Starting from 2 founders we have formed a population of 150 (Figure 5A) and 300 (Figure 5B) genotypes with 1000 single nuceotide polymorphism (SNPs) at 3 chromosomes each and carried this population through 200 generations of random mating and 100 generations of phenotypic selection based on a complex trait (300 QTL at random locations on each chromosome) with 0.5 heritability generated based on the infinitesimal model. Starting from this initial population, we have simulated 10 rounds of PS, and 20 rounds of GS and GM (assuming one cycle of PS and two cycles of GS and GM per year). For GS and GM, the marker effects were estimated from data once per year. The results of 10 replication of this simulation with selection intensity 10% (PS1, GS1) and 20% (PS2, GS2) for PS and GS; Efficient GS (GSeff); and GM with λ_2_ = 0, 5, 10 (GM1, GM2, GM3). In this simulation study, there is a clear advantage of using GM as a breeding method since the gains attained by this method are higher as compared to the other methods, especially in the long run (see Figures 5A,B).

## 4. Discussion

In this article, we have proposed a mating methodology for breeding based on optimal genomic determination of mating plans. In GS, the breeding value is predicted using a statistical model based on phenotypes and whole genome marker data (obtained within an experiment that is repeated in every few cycles, blue arrows in Figure 1 selection is based on GEBVs. Our approach can be contrasted with the selection approach where only proportional contributions of parents to the progeny that balances gain and inbreeding are sought. A major novelty in GM approach as compared to the other methods (Kinghorn, 1998; Shepherd and Kinghorn, 1998) is the utilization of a genomically estimated within cross-variances (usefulness) in the objective function along with genetic gains and inbreeding. Although similar to GS in its information requirements, our approach offers more complete utilization of the genotypic and phenotypic information.

We did not explore any alternatives to our mating optimization algorithm, but similar evolutionary algorithms like differential evolution, particle swarm, tabu search, and simulated annealing or hill climbing methods like the exchange method can be useful to solve this problem. As stated by other authors Kinghorn (2011) and Pryce et al. (2012), the mate selection problem has two independent components: a mate selection index (MSI), i.e., the optimization function and a mate selection algorithm that can be used to optimize the MSI. In our article, we have provided new approaches to both of these components: First, the MSI we have used differed from previous authors and included terms for gain, variance and inbreeding, and secondly, we have adopted a genetic algorithm that can find good solutions. The objective function that we have proposed only uses additive marker effects, but would be desirable to extend the objective function to include effects and variances related to dominance, and interactions.

As opposed to the continuous parentage contribution proportions solutions in the GS method, the mating method gives discrete solutions. That is to say, the solutions of the mating algorithm are the list of parent mates of the progeny (Figure 4). Additionally, while using GS method, there is no real guideline for choosing an optimal solution among the possibilities on the frontier curve. Conversely, since the mating algorithm is discrete and the number of genotypes contributing to the next generation increase starting from one as we increase the λ_2_, we can identify a point to operate on this surface by slowly increasing the λ_2_ until a desired minimum number of genotypes are included in the solution. This is the method we have used in our long term simulations.

We claim that GM uses genomic information more completely than the recently proposed GS and reinforces mating complementary individuals. In an scenario where a set of individuals with their markers and related marker effects are given in a breeding population, the GM approach gives a list of mates that should be crossed for obtaining the next breeding population and, unlike selection methods, the proposed GM approach does not exclude the possibility of contribution of all individuals as parents. A cross-variance term is included in the objective function along with genetic gains and inbreeding to account for potential benefits from including mates with higher estimated genetic variance. To this end, we provide a method that uses marker effect estimates to estimate within cross-variances assuming independence among loci and additive effects. Using simulations, we have compared the long range performance of GM to PS, GS and an optimal parentage contribution approach. Results from these simulations point to the viability and efficiency of genomic mating for breeding complex traits.

In practice, mating designs will be different in plant and animal breeding and will reflect the constraints that are related to many factors such the reproduction properties of the species, other biological and logistical constraints. In plant breeding, the most important factors affecting the choices of mating designs are: (i) the type of pollination (self vs. cross-pollinated), (ii) control of pollination (natural or artificial, genetic control of pollination or the presence of male-sterility system), and (iii) the size of the population required. In animal breeding, mating designs will reflect (i) the number of males and more importantly the number of females in the breeding population, (ii) constraints related the female reproduction. Breeders also use different hierarchical structures, such as half-, full-sib family, and individuals within family, in the breeding population. The simulations herein are limited in terms of reflecting real life plant and animal breeding scenarios, more research, simulations and real experiments should be conducted to fully evaluate this methodology.

The design of training populations for GS models attained a lot of attention recently (Rincent et al., 2012; Akdemir et al., 2015; Isidro et al., 2015). This approach is promising because any gain in accuracy or any reduction in experimental costs that can be obtained by carefully designing the training populations will proportionately be realized as gains. It is perceivable to have two distinct populations, one for the training models and the other the breeding. However, this is rare. The simulations in this manuscript have used a single population for breeding and training GS models akin to many real life realizations of GS. The point is that the models used for GS and GM might have different accuracies since they use the most recent experiments' data obtained from different populations and this might affect the accuracies of the models. Therefore, there might be a component of GM that allows better designs in terms of model accuracies that is reflected as the higher gains in GM as compared to GS. We expect such an effect since proposed method controls inbreeding and co-ancestry in the breeding population. We have not fully explored segregation of the gains into such components. Nevertheless, in addition to being useful for generating a breeding population to be used as a basis improvement and development of potential varieties, GM can be useful to provide information on the genetic control of the character under investigation; provide estimates of genetic gain and provide information for evaluating the parents used in the breeding program (Nduwumuremyi et al., 2013).

There is an intrinsic limit to the amount of selfing or crosses of closely related lines in GM. Although it is hard to imagine that this is what is done in practice, theoretically, leaving the decision to a “roulette wheel” assignment of parents as mates as in the selection approach might lead to too much inbreeding. For example, if the parental contribution proportion of a parent is 0.50, then we expect to have 25% obtained by selfing this parent. Genomic mating allows a better control of inbreeding by completely controlling who mates with whom.

It should be pointed out that GS and PS are often used in conjunction with methods that do seek to optimize the combination of parents to be mated. The most prominent case of hybrid breeding, e.g., in maize, where parents from two opposing heterotic pools are selected to produce the best hybrids, using estimates of general and specific combining ability. There is ample literature on this, e.g., Schrag et al. (2009) and Technow et al. (2012). The main concern of the current article is improvement of complex traits by recurrent crosses for out-crossing plants as in mass selection to increase the frequency of desirable genes in the base population. Selection of mates from heterotic groups or pools of inbred lines are related problems, however they are outside the scope of this article.

In our examples, we assumed an infinitesimal model for the simulated trait with many small effects throughout the genome (300 QTL on each of the 3 chromosomes and the effects were generated from a zero centered normal distribution); we have only made use of the ridge regression (rr-BLUP) model to estimate the marker effects. But the reader should note that the results will be always affected by the features of simulated data. Trait architecture and prediction model will be important components that will affect the relative performance of GM. It is also known that in genomic prediction many sparse methods like Bayesian lasso, etc,…would benefit for traits controlled by few QTL, while rr-BLUP (or equivalently G-BLUP) favors traits without major gene. Moreover, in many breeding programs, the interest is on improvement of multiple traits at the same time. Marker effects estimated based on the trait of interest as well as other correlated traits can be integrated into an objective function. We did not explore any of these scenarios since they would not fit into the scope of this current article. These are important issues that we hope to address with subsequent research and publications.

Finally, It should be clear from our discussions we understand that breeding is a complex human endeavor influences by an enourmous number of factors and we do not intend to propose GM as a replacement of PS, GS or any other accepted and widely used breeding principles. Genomic mating should be seen as an additional informative tool for breeders providing suggestions about design and management of their breeding programs, yet from another perspective.

## Author contributions

DA: Conception or design of the work, programs and simulations, drafting the article, critical revision of the article. JS: Drafting the article, critical revision of the article.

### Conflict of interest statement

The authors declare that the research was conducted in the absence of any commercial or financial relationships that could be construed as a potential conflict of interest.

## Supplementary material

### Supplemental data

Data Sheet 1 includes the code used for the simulating the data and applying the breeding schemes. Genetic data is simulated using the CRAN package “hypred” (Technow, 2014). Mixed model software is publicly available via CRAN (Package EMMREML) (Akdemir and Godfrey, 2015). The rest of the software were written using C++ and R. The code is available from the authors.
