# Supplementary material for: Efficient Breeding by Genomic Mating
Source: Front Genet. 2016 Nov 29;7:210. doi: 10.3389/fgene.2016.00210 (PMC5126051; doi:10.3389/fgene.2016.00210)
Supplement: Supplementary file 1 [file DataSheet1.pdf]

```
####functins for applying breeding scheme (use of hyped and EMMREML
packages)
###Will not work without additional code from the corresponding
author.
breedingcycles.C0<-function(lambda, complexity, populationsize,
selectionintensity){

  library(hypred)
  N=populationsize

  genome <- hypredGenome(num.chr =3,
                         len.chr = rep(1,3), num.snp.chr=1000,
num.blocks.chr = 100)
  summary(genome)
  map <-slot(genome, "pos.snp")




  qtl.ids1 <- sort(sample(1:1000, complexity))
  qtl.ids2 <- sort(sample(1001:2000, complexity))
  qtl.ids3 <- sort(sample(2001:3000, complexity))

  qtl.ids<-c(qtl.ids1, qtl.ids2, qtl.ids3)
  qtl.dom.ids <- sort(c(sample(qtl.ids1, ceiling(complexity*.1)),
                        sample(qtl.ids2, ceiling(complexity*.1)) ,
                        sample(qtl.ids3, ceiling(complexity*.1)) ))
  per.mar.ids <- qtl.ids
  genome <- hypredNewQTL(genome,
                         new.id.add = qtl.ids,
                         new.id.dom = qtl.dom.ids,
                         new.id.per.mar = per.mar.ids,
                         new.eff.add =  sqrt(1/(.
9*length(qtl.ids)))*rnorm(length(qtl.ids)),
                         new.eff.dom = sqrt(1/(.
1*length(qtl.ids)))*rnorm(length(qtl.dom.ids))
  )
  summary(genome)

  founders<-hypredFounder(genome,
                            prob.snp = 1)
  founder1 <- founders[1,]#hypredFounder(genome,prob.snp = 1)[1, ]
  founder2 <- founders[2,]#hypredFounder(genome,prob.snp = 1)[2, ]



  F2 <- matrix(nrow = N * 2,ncol = 3000)
```

```
for(i in 1:(N*2))
{
  F2[i, ] <- hypredRecombine(genome,
                             genomeA = founder1,
                             genomeB = founder2,
                             mutate = TRUE,
                             mutation.rate.snp = 2.5 * 10^-5,
                             mutation.rate.qtl = 2.5 * 10^-5,
                             block = sample(c(TRUE,FALSE), 1))
}


design.M<- hypredCode(genome,
                      genotypes = F2,
                      DH = FALSE,
                      type = "012")




#####F3 is obtained by selfing F2

F3 <- F2 ## identical to the F2 at start
F3.temp <- matrix(nrow = N*2, ncol = 3000)


gameteIndex1 <- 1 ## indexing variables
gameteIndex2 <- 2
for(indiv in 1 : N) ## loop over individuals
{
  ## gamete 1
  F3.temp[gameteIndex1,] <-
    hypredRecombine(genome,
                    genomeA = F3[gameteIndex1,],
                    genomeB = F3[gameteIndex2,],
                    mutate = TRUE,
                    mutation.rate.snp = 2.5 * 10^(-5),
                    mutation.rate.qtl = 2.5 * 10^(-5),
                    block = sample(c(TRUE,FALSE), 1))
  ## gamete 2
  F3.temp[gameteIndex2,] <-
    hypredRecombine(genome,
                    genomeA = F3[gameteIndex1,],
                    genomeB = F3[gameteIndex2,],
                    mutate = TRUE,
                    mutation.rate.snp = 2.5 * 10^(-5),
                    mutation.rate.qtl = 2.5 * 10^(-5),
                    block = sample(c(TRUE,FALSE), 1))
  ## increment to next individual
  gameteIndex1 <- gameteIndex1 + 2
```

```
    gameteIndex2 <- gameteIndex2 + 2
  } ## end for N
  ## permutate
  F3 <- F3.temp


  design.M3<- hypredCode(genome,
                         genotypes = F3,
                         DH = FALSE,
                         type = "012")




  ######Generate Phenotype and Genotype data
  ########Build marker based model, estimate marker effects
  g.value <- hypredTruePerformance(genome,
                                   F3,DH=FALSE)
  var.env <- var(g.value)
  phen.value <- g.value + rnorm(N, 0,  sqrt(lambda)*sqrt(var.env))




  library(EMMREML)

  model<-emmreml(y=phen.value, X=matrix(1,
nrow=length(phen.value),ncol=1), Z=design.M3-1,
K=diag(ncol(design.M3)))
  model$uhat
  #######Phenotypic selection



  index.individuals <- which(phen.value >= quantile(phen.value, 1-.8))
  llindex<-.01
  while (length(index.individuals)<10){
    index.individuals <- which(phen.value >= quantile(phen.value, 1-
(.8+llindex)))
    llindex=llindex+.01
  }
  selected<-c()
  for (i in 1:length(index.individuals)){
    index.row1 <- index.individuals[i] * 2 - 1
    index.row2 <- index.individuals[i] * 2
    selected <- rbind(selected,F3[c(index.row1, index.row2), ])
  }
  Npar<-length(index.individuals)
  #print(Npar)
```

```
  ######Inititial Population from selected parents
  S1 <- matrix(nrow = N*2, ncol = 3000) ## identical to the F2 at
start
  S1.temp <- matrix(nrow = N*2, ncol = 3000)

  gameteIndex1 <- 1 ## indexing variables
  gameteIndex2 <- 2
  for(indiv in 1 : N) ## loop over individuals
  {
    ## gamete 1
    par1<-sample(seq(1,(Npar*2),by=2),1)
    par2<-sample(seq(1,(Npar*2),by=2),1)
    S1.temp[gameteIndex1,] <-
      hypredRecombine(genome,
                      genomeA = selected[par1,],
                      genomeB = selected[par1+1,],
                      mutate = TRUE,
                      mutation.rate.snp = 2.5 * 10^(-5),
                      mutation.rate.qtl = 2.5 * 10^(-5),
                      block = sample(c(TRUE,FALSE), 1))
    ## gamete 2
    S1.temp[gameteIndex2,] <-
      hypredRecombine(genome,
                      genomeA = selected[par2,],
                      genomeB = selected[par2+1,],
                      mutate = TRUE,
                      mutation.rate.snp = 2.5 * 10^(-5),
                      mutation.rate.qtl = 2.5 * 10^(-5),
                      block = sample(c(TRUE,FALSE), 1))
    ## increment to next individual
    gameteIndex1 <- gameteIndex1 + 2
    gameteIndex2 <- gameteIndex2 + 2
  } ## end for N

  S1 <- S1.temp


  design.S1<- hypredCode(genome,
                         genotypes = S1,
                         DH = FALSE,
                         type = "012")
  return(list(genome=genome, gametes=S1,markermatrix=design.S1,
model=model, var.env=var.env))

}



breedingcycles.C1PS<-function(initpop,selectionintensity=.1, var.env,
```

```
lambda){




  S2 <- initpop$gametes## identical to the F2 at start
  genome<-initpop$genome
  N<-nrow(S2)/2
  nmarkers<-ncol(S2)
  S2.temp <- matrix(nrow = N*2, ncol = nmarkers)


  gameteIndex1 <- 1 ## indexing variables
  gameteIndex2 <- 2
  for(indiv in 1 : N) ## loop over individuals
  {
    ## gamete 1
    S2.temp[gameteIndex1,] <-
      hypredRecombine(genome,
                      genomeA = S2[gameteIndex1,],
                      genomeB = S2[gameteIndex2,],
                      mutate = TRUE,
                      mutation.rate.snp = 2.5 * 10^(-5),
                      mutation.rate.qtl = 2.5 * 10^(-5),
                      block = sample(c(TRUE,FALSE), 1))
    ## gamete 2
    S2.temp[gameteIndex2,] <-
      hypredRecombine(genome,
                      genomeA = S2[gameteIndex1,],
                      genomeB = S2[gameteIndex2,],
                      mutate = TRUE,
                      mutation.rate.snp = 2.5 * 10^(-5),
                      mutation.rate.qtl = 2.5 * 10^(-5),
                      block = sample(c(TRUE,FALSE), 1))
    ## increment to next individual
    gameteIndex1 <- gameteIndex1 + 2
    gameteIndex2 <- gameteIndex2 + 2
  } ## end for N
  ## permutate
  S2 <- S2.temp



  ######Generate Phenotype and Genotype data
  ########Build marker based model, estimate marker effects
  g.value <- hypredTruePerformance(genome,
                                   S2,DH=FALSE)
  phen.value <- g.value + rnorm(N, 0,  sqrt(lambda)*sqrt(var.env))
```

```
  design.S2<- hypredCode(genome,
                         genotypes = S2,
                         DH = FALSE,
                         type = "012")
  library(EMMREML)

  model<-emmreml(y=phen.value, X=matrix(1,
nrow=length(phen.value),ncol=1), Z=design.S2-1,
K=diag(ncol(design.S2)))

  #######Phenotypic selection
  cutoffPS<-quantile(phen.value, 1- selectionintensity)

  index.individuals <- which(phen.value >= cutoffPS)
  llindex<-.01
  while (length(index.individuals)<10){
    index.individuals <- which(phen.value >= quantile(phen.value, 1-
(selectionintensity+llindex)))
    llindex=llindex+.01
  }

  selected<-c()
  for (i in 1:length(index.individuals)){
    index.row1 <- index.individuals[i] * 2 - 1
    index.row2 <- index.individuals[i] * 2
    selected <- rbind(selected,S2[c(index.row1, index.row2), ])
  }
  Npar<-length(index.individuals)
  #print(Npar)
  ######Inititial Population from selected parents
  S1 <- matrix(nrow = N*2, ncol = nmarkers) ## identical to the F2 at
start
  S1.temp <- matrix(nrow = N*2, ncol = nmarkers)

  gameteIndex1 <- 1 ## indexing variables
  gameteIndex2 <- 2
  for(indiv in 1 : N) ## loop over individuals
  {
    ## gamete 1
    par1<-sample(seq(1,(Npar*2),by=2),1)
    par2<-sample(seq(1,(Npar*2),by=2),1)
    S1.temp[gameteIndex1,] <-
      hypredRecombine(genome,
                      genomeA = selected[par1,],
                      genomeB = selected[par1+1,],
                      mutate = TRUE,
                      mutation.rate.snp = 2.5 * 10^(-5),
                      mutation.rate.qtl = 2.5 * 10^(-5),
                      block = sample(c(TRUE,FALSE), 1))
```

```
    ## gamete 2
    S1.temp[gameteIndex2,] <-
      hypredRecombine(genome,
                      genomeA = selected[par2,],
                      genomeB = selected[par2+1,],
                      mutate = TRUE,
                      mutation.rate.snp = 2.5 * 10^(-5),
                      mutation.rate.qtl = 2.5 * 10^(-5),
                      block = sample(c(TRUE,FALSE), 1))
    ## increment to next individual
    gameteIndex1 <- gameteIndex1 + 2
    gameteIndex2 <- gameteIndex2 + 2
  } ## end for N

  S1 <- S1.temp


  design.S1<- hypredCode(genome,
                         genotypes = S1,
                         DH = FALSE,
                         type = "012")

  return(list(genome=genome, gametes=S1,markermatrix=design.S1,
model=model))

}





breedingcycles.C1GS<-function(initpop, selectionintensity=.
1,modelupdate=T, var.env, lambda){


  S2 <- initpop$gametes## identical to the F2 at start
  genome<-initpop$genome
  model<-initpop$model
  N<-nrow(S2)/2
  nmarkers<-ncol(S2)
  design.S2<- hypredCode(genome,
                         genotypes = S2,
                         DH = FALSE,
                         type = "012")

  GEBVsS2<-(design.S2-1)%*%model$uhat


  #######Genomic selection
```

```
  index.individuals <- which(GEBVsS2 >= quantile(GEBVsS2, 1-
selectionintensity))

  llindex<-.01
  while (length(index.individuals)<10){
    index.individuals <- which(GEBVsS2 >= quantile(GEBVsS2, 1-
(selectionintensity+llindex)))

    llindex=llindex+.01
  }



  selected<-c()
  for (i in 1:length(index.individuals)){
    index.row1 <- index.individuals[i] * 2 - 1
    index.row2 <- index.individuals[i] * 2
    selected <- rbind(selected,S2[c(index.row1, index.row2), ])
  }
  Npar<-length(index.individuals)
  #print(Npar)

  ######Inititial Population from selected parents
  S1 <- matrix(nrow = N*2, ncol = nmarkers) ## identical to the F2 at
start
  S1.temp <- matrix(nrow = N*2, ncol = nmarkers)

  gameteIndex1 <- 1 ## indexing variables
  gameteIndex2 <- 2
  for(indiv in 1 : N) ## loop over individuals
  {
    ## gamete 1
    par1<-sample(seq(1,(Npar*2),by=2),1)
    par2<-sample(seq(1,(Npar*2),by=2),1)
    S1.temp[gameteIndex1,] <-
      hypredRecombine(genome,
                      genomeA = selected[par1,],
                      genomeB = selected[par1+1,],
                      mutate = TRUE,
                      mutation.rate.snp = 2.5 * 10^(-5),
                      mutation.rate.qtl = 2.5 * 10^(-5),
                      block = sample(c(TRUE,FALSE), 1))
    ## gamete 2
    S1.temp[gameteIndex2,] <-
      hypredRecombine(genome,
                      genomeA = selected[par2,],
                      genomeB = selected[par2+1,],
                      mutate = TRUE,
                      mutation.rate.snp = 2.5 * 10^(-5),
                      mutation.rate.qtl = 2.5 * 10^(-5),
```

```
                      block = sample(c(TRUE,FALSE), 1))
    ## increment to next individual
    gameteIndex1 <- gameteIndex1 + 2
    gameteIndex2 <- gameteIndex2 + 2
  } ## end for N

  S1 <- S1.temp


  design.S1<- hypredCode(genome,
                         genotypes = S1,
                         DH = FALSE,
                         type = "012")
  if (modelupdate){
    ######Generate Phenotype and Genotype data
    ########Build marker based model, estimate marker effects
    g.value <- hypredTruePerformance(genome,
                                     S1,DH=FALSE)

    phen.value <- g.value + rnorm(N, 0,  sqrt(lambda)*sqrt(var.env))




    library(EMMREML)
    print((design.S1-1)[1:5,1:5])
    model<-emmreml(y=phen.value, X=matrix(1,
nrow=length(phen.value),ncol=1), Z=design.S1-1,
K=diag(ncol(design.S1)))
  }
  return(list(genome=genome, gametes=S1,markermatrix=design.S1,
model=model))

}


#####





######################################################################
##################

breedingcycles.C1GSMatingStrategy<-
function(initpop,impvar,impforinbreed,minparents,
impinbreedstepsize,modelupdate, var.env, lambda,npopGA, nitGA,
plotiters , mc.cores, nelite, mutprob){
```

```
  ######Cycle  (by selfing s1)
  ######Generate Phenotype and Genotype data
  ########Build marker based model, estimate marker effects
  #######Phenotypic selection


  S2 <- initpop$gametes## identical to the F2 at start
  genome<-initpop$genome
  model<-initpop$model
  N<-nrow(S2)/2
  nmarkers<-ncol(S2)
  design.S2<- hypredCode(genome,
                       genotypes = S2,
                       DH = FALSE,
                       type = "012")
  Amat2<-Kmatfunc( design.S2-1)
  GEBVsS2<-(design.S2-1)%*%model$uhat


  solGA<-getGaSolutions(design.S2-1, Amat2, model$uhat,
impvar=impvar, impforinbreed=impforinbreed,minparents=minparents,
impinbreedstepsize=impinbreedstepsize,npopGA=npopGA, nitGA=nitGA,
plotiters=plotiters, mc.cores=mc.cores,nelite=nelite, mutprob=mutprob)

  ######Inititial Population from selected parents
  S1 <- matrix(nrow = N*2, ncol = nmarkers) ## identical to the F2 at
start
  S1.temp <- matrix(nrow = N*2, ncol = nmarkers)
  #print(solGA)
  #print(order(GEBVsS2, decreasing=T))
  gameteIndex1 <- 1 ## indexing variables
  gameteIndex2 <- 2
  for(indiv in 1 : N) ## loop over individuals
  {
    ## gamete 1
    par1<-2*solGA[indiv,1]-1
    par2<-2*solGA[indiv,2]-1
    # print(c(par1,par2))
    S1.temp[gameteIndex1,] <-
      hypredRecombine(genome,
                      genomeA = S2[par1,],
                      genomeB = S2[par1+1,],
                      mutate = TRUE,
                      mutation.rate.snp = 2.5 * 10^(-5),
                      mutation.rate.qtl = 2.5 * 10^(-5),
                      block = sample(c(TRUE,FALSE), 1))
    ## gamete 2
    S1.temp[gameteIndex2,] <-
      hypredRecombine(genome,
```

```
                      genomeA = S2[par2,],
                      genomeB = S2[par2+1,],
                      mutate = TRUE,
                      mutation.rate.snp = 2.5 * 10^(-5),
                      mutation.rate.qtl = 2.5 * 10^(-5),
                      block = sample(c(TRUE,FALSE), 1))
    ## increment to next individual
    gameteIndex1 <- gameteIndex1 + 2
    gameteIndex2 <- gameteIndex2 + 2
  } ## end for N

  S1 <- S1.temp


  design.S1<- hypredCode(genome,
                         genotypes = S1,
                         DH = FALSE,
                         type = "012")
  if (modelupdate){
    ######Generate Phenotype and Genotype data
    ########Build marker based model, estimate marker effects
    g.value <- hypredTruePerformance(genome,
                                     S1,DH=FALSE)
    phen.value <- g.value + rnorm(N, 0,  sqrt(lambda)*sqrt(var.env))




    library(EMMREML)

    model<-emmreml(y=phen.value, X=matrix(1,
nrow=length(phen.value),ncol=1), Z=design.S1-1,
K=diag(ncol(design.S1)))
  }
  return(list(genome=genome, gametes=S1,markermatrix=design.S1,
model=model, solGA=solGA))

}

breedingcycles.C1GSEFF0<-function(initpop, qcut,modelupdate=T,
var.env, lambda){

  ######Cycle  (by selfing s1)
  ######Generate Phenotype and Genotype data
  ########Build marker based model, estimate marker effects
  #######Phenotypic selection


  S2 <- initpop$gametes## identical to the F2 at start
  genome<-initpop$genome
```

```
  model<-initpop$model
  N<-nrow(S2)/2
  nmarkers<-ncol(S2)
  design.S2<- hypredCode(genome,
                         genotypes = S2,
                         DH = FALSE,
                         type = "012")

  GEBVsS2<-(design.S2-1)%*%model$uhat

  Amat2<-Kmatfunc( design.S2-1)
  colnames(Amat2)<-rownames(Amat2)<-names(GEBVsS2)<-paste("l",
1:length(GEBVsS2),sep="")

  eigAmat2<-eigen(Amat2, symmetric=T)
  eigAmat2$values[eigAmat2$values<1e-6]<-1e-6
  Amat2<- eigAmat2$vectors%*%diag(eigAmat2$values)%*
%t(eigAmat2$vectors)
  colnames(Amat2)<-rownames(Amat2)<-names(GEBVsS2)<-paste("l",
1:length(GEBVsS2),sep="")

  outefffront<- efficientfrontier(Amat=Amat2, gebvs=GEBVsS2,
ul=1,npointsforefffront=npointsforefffront)

  maxret<-max(outefffront[,N+2])
  minret<-min(outefffront[,N+2])
  minpoint<-which.min(abs(qcut*(maxret-minret)+minret-outefffront[,N
+2]))
  impforbreedest<-outefffront[minpoint,N+1]
  eff.optimal.point <-outefffront[minpoint,1:N]


  index.individuals <- which(GEBVsS2 >= quantile(GEBVsS2, 0))
  selected<-c()
  for (i in 1:length(index.individuals)){
    index.row1 <- index.individuals[i] * 2 - 1
    index.row2 <- index.individuals[i] * 2
    selected <- rbind(selected,S2[c(index.row1, index.row2), ])
  }
  Npar<-length(index.individuals)

  # print(length(eff.optimal.point[eff.optimal.point>0]))
  ######Initittial Population from selected parents
  S1 <- matrix(nrow = N*2, ncol = nmarkers) ## identical to the F2 at
start
  S1.temp <- matrix(nrow = N*2, ncol = nmarkers)

  gameteIndex1 <- 1 ## indexing variables
  gameteIndex2 <- 2
  for(indiv in 1 : N) ## loop over individuals
```

```
  {
    ## gamete 1

    par1<-sample(seq(1,(Npar*2),by=2),size=1,prob=eff.optimal.point)
    par2<-sample(seq(1,(Npar*2),by=2),size=1,prob=eff.optimal.point)
    S1.temp[gameteIndex1,] <-
      hypredRecombine(genome,
                      genomeA = selected[par1,],
                      genomeB = selected[par1+1,],
                      mutate = TRUE,
                      mutation.rate.snp = 2.5 * 10^(-5),
                      mutation.rate.qtl = 2.5 * 10^(-5),
                      block = sample(c(TRUE,FALSE), 1))
    ## gamete 2
    S1.temp[gameteIndex2,] <-
      hypredRecombine(genome,
                      genomeA = selected[par2,],
                      genomeB = selected[par2+1,],
                      mutate = TRUE,
                      mutation.rate.snp = 2.5 * 10^(-5),
                      mutation.rate.qtl = 2.5 * 10^(-5),
                      block = sample(c(TRUE,FALSE), 1))
    ## increment to next individual
    gameteIndex1 <- gameteIndex1 + 2
    gameteIndex2 <- gameteIndex2 + 2
  } ## end for N

  S1 <- S1.temp


  design.S1<- hypredCode(genome,
                         genotypes = S1,
                         DH = FALSE,
                         type = "012")
  if (modelupdate){
    ######Generate Phenotype and Genotype data
    ########Build marker based model, estimate marker effects
    g.value <- hypredTruePerformance(genome,
                                     S1,DH=FALSE)
    phen.value <- g.value + rnorm(N, 0,  sqrt(lambda)*sqrt(var.env))

    library(EMMREML)

    model<-emmreml(y=phen.value, X=matrix(1,
nrow=length(phen.value),ncol=1), Z=design.S1-1,
K=diag(ncol(design.S1)))
  }
  return(list(genome=genome, gametes=S1,markermatrix=design.S1,
model=model,prob=eff.optimal.point))
```

```
}
```
